# Factors associated with acute kidney injury (AKI) and mortality in COVID-19 patients in a Sub-Saharan African intensive care unit: a single-center prospective study

**DOI:** 10.1080/0886022X.2023.2263583

**Published:** 2023-10-23

**Authors:** Yannick Nlandu, Jean-Robert Makulo, Marie Essig, Ernest Sumaili, Aimé Lumaka, Yannick Engole, Marie-France Mboliasa, Vieux Mokoli, Trésor Tshiswaka, Aliocha Nkodila, Justine Bukabau, Augustin Longo, François Kajingulu, Chantal Zinga, Nazaire Nseka

**Affiliations:** a Nephrology Unit, Kinshasa University Hospital, University of Kinshasa, Kinshasa, Democratic Republic of Congo; b Intensive Care Unit, Centre Médical de Kinshasa, Kinshasa, Democratic Republic of Congo; c Nephrology Department, Ambroise Paré Hospital, AP-HP University Paris-Saclay, Boulogne-Billancourt, France; d Center for Human Genetics, Department of Pediatrics, University of Kinshasa, Kinshasa, Democratic Republic of Congo; e Department of Family Medicine and Primary Care, Protestant University in Congo, Kinshasa, Democratic Republic of Congo

**Keywords:** AKI, black people, covid-19, risk factors, mortality

## Abstract

**Introduction:**

Acute kidney injury (AKI) is a complication of severe coronavirus disease 2019 (COVID-19). Kidney damage associated with COVID-19 could take specific features due to environmental and socio-cultural factors. This study evaluates the incidence of AKI, the associated factors, and mortality in COVID-19 patients in a Sub-Saharan African intensive care unit.

**Methods:**

In a prospective cohort study conducted in the intensive care unit (ICU) of the Centre Médical de Kinshasa (CMK), consecutive patients admitted for COVID-19 were screened for the presence of AKI between 27 March, 2020 and 27 January 2022. AKI was defined according to Kidney Disease Improving Global Outcomes (KDIGO) guidelines. The primary outcome was occurrence of AKI. The secondary outcome was 48 days’ mortality and recovery of the renal function at intensive care unit (ICU) discharge. Survival (time-to death) curves were built using the Kaplan Meier methods. Multivariate analyses were performed by logistic regression to identify factors associated with AKI and Cox regression to explore the association between AKI and in-hospital mortality. The significance level of the *p*-value was set at 0.05.

**Results:**

The median(IQR) sequential organ failure assessment score (SOFA) score and mean age of patients (215) including in our cohort were respectively 3(2-4) and 58.9 ± 14.9 years. The incidence of AKI was 28.4% with stages 1, 2, or 3 AKI accounted for 39.3%, 11.5%, and 49.2%, respectively. Hemodialysis was required in 16 out 215 (7.4%) patients. Dyspnea (adjusted odds ratio (aOR):2.27 [1.1–-4.57] *p* = 0.021), SOFA ≥5 (aOR:3.11[1.29–7.53] *p* = 0.012), AST/ALT ratio (aOR: 1.53 [1.09–1.79] *p* = 0.015), N/L ratio (aOR:2.09 [1.09–3.20] *p* = 0.016), mechanical ventilation (aOR: 3.20 [1.66–10.51] *p* = 0.005) and Amikacin (aOR: 2.91 [1.37–6.18] *p* = 0.006) were the main factors associated with AKI. Patients with AKI had a mortality rate of 52.5% and 67.2% of the survivors did not recover kidney function at the end of hospitalization. Adjusted Cox regression analysis revealed that COVID-19-associated AKI was independently associated with in-hospital death (HR:2.96 [1.93–4.65] *p* = 0.013) compared to non-AKI patients.

**Conclusions:**

AKI was present in three out of ten COVID-19 patients. The most significant factors associated with AKI were dyspnea, SOFA ≥ 5, AST/ALT and N/L ratio, mechanical ventilation and Amikacin. AKI has been associated with an almost threefold increase in overall mortality and seven out of ten survivors did not recover kidney function after AKI.

## Introduction

In December 2019, the world started facing a new pandemic associated with the coronavirus disease 2019 (COVID-19). This severe acute respiratory syndrome coronavirus (SARS-COV-2) has infected more than 500 million people around the world with more than six million deaths, fueled by the different waves of infection carried by the appearance of many variants that are sometimes more contagious as illustrated by the delta variant [1].

Initially described mainly as a respiratory pathology, SARS-COV-2 infection has proven to be a more serious disease that can be responsible for multi-visceral damage, including kidney damage [2,3]. Although the frequency of acute kidney injury (AKI), with or without proteinuria, was negligible in the first studies on COVID-19, recent series report a higher frequency, particularly in patients admitted to the intensive care unit (ICU) [4–6]. Gradually, the various risk factors of acute kidney injury associated with COVID-19 (COV-AKI) are being identified, including patient-specific factors (age, ethnicity, comorbidities [chronic kidney disease (CKD), diabetes, hypertension…], genetic predisposition such as the the presence of apolipoprotein 1 (APOL-1) risk alleles, the severity of the disease well reflected by the levels of inflammatory markers that are indirect witnesses of the cytokine storm phenomenon or by hypoxemia, as well as exposure to certain therapeutic interventions (mechanical ventilation, vasopressors, antibiotics, etc.) [7]. At the same time, COV-AKI has been shown to be a predictor of mortality [8] and for survivors, renal recovery varies according to the operational definition used in different studies, but remains lower when compared with other causes of AKI [9]. AKI was found to be also a predictor for readmission and mortality in COVID-19 patients discharged early from the hospital [10].

Africa has also been affected by COVID-19, although the predicted magnitude was not consistent with reality [11]. Multiple case report and small size studies reported an association between COVID-19 and kidney damage. Diana et al. were the first to describe AKI in a large cohort of 1102 COVID-19 patients in the Republic of South Africa [12]. These authors observed a frequency of COV-AKI of 33.9% and identified risk factors similar to those reported in Western and Asian literature [12]. In the Democratic Republic of the Congo, it is likely that COVID-19-associated kidney damage could exhibit specific characteristics because of the African genetic background on one hand, and the local environmental and sociocultural factors on the other hand. For instance, the prevalence of APOL-1 risk alleles for kidney disease, is high in this population [13]. This high prevalence could exacerbate the severity of AKI [14]. Regarding the environmental and sociocultural factors, the coexistence of infectious diseases such as malaria and the cultural attachment to traditional medicine, could further modify the presentation of AKI during COVID-19 in African communities. This study aimed at determining the incidence of COV-AKI, the associated factors, and the outcome in a cohort of Sub-Saharan African.

## Methods

### Study design, setting, and population

This prospective, observational, cohort study was conducted at the ICU of the Centre Médical de Kinshasa (CMK), a private facility appointed by the Ministry of Public Health as one of the few reference centers for the management of the COVID-19. CMK is located in the commune of Gombe, the epicenter of the COVID-19 disease in Kinshasa, capital of the Democratic Republic of Congo (DRC). Most of the patients admitted to the CMK had healthcare coverage fully provided by the employer, whereas few were nonaffiliated and payed out of their own pocket. At the time of COVID-19, CMK had two intensive care units, one of which was specially dedicated to COVID-19 patients with 15 intensive care unit beds and a ventilator to bed ratio of 1:1. The medical team included two nephrologists who were responsible for prescribing and monitoring hemodialysis sessions. The COVID-19 intensive care unit was equipped with one hemodialysis machine. Intermittent hemodialysis was the only one modality of renal replacement therapy.

To be recruited, patients had been at least 18-year-old, have a positive reverse transcription polymerase chain reaction (RT-PCR) COVID-19 test, and hospitalized and/or followed up at the ICU of the CMK during the first 4 COVID-19 waves from 27 March 2020 to 27 January 2022. Wave was defined according to the Congolese Technical Secretariat of the Multisectoral COVID-19 response committee (First wave: week 16/2020–week 34/2020; second wave: week 47/2020–week 13/2021; third wave: week 20/2021-week 32/2021; fourth wave: week 45/2021–week 2/2022) [14]. At the time of the study, the Democratic Republic of Congo had one of the lowest COVID-19 vaccination rates, with less than 4% of the total population fully vaccinated [14]. Only patients who demonstrated signs of moderate, severe and critical illness were admitted to hospital and systematically treated with macrolides and chloroquine in accordance with national guidelines. The positive experience of using this conventional treatment in many African countries, including the DRC, justifies the continuation of this therapeutic protocol for all 4 waves [14,15]. The sample size was not predetermined and all patients meeting the inclusion criteria were recruited consecutively. Patients undergoing chronic dialysis (hemodialysis or peritoneal dialysis) and those with less than two serum creatinine (SCr) measurements were excluded.

### Data collection

The clinical (demographic characteristics [age, gender and race], the existence of any chronic conditions [hypertension, diabetes mellitus, obesity, chronic kidney disease (CKD)], vital signs (temperature, respiratory rate [RR], and blood oxygen saturation [BOS]), biological (hemoglobin [Hb], hematocrit [Hte], inflammatory markers (white blood cells [WBC], neutrophils, lymphocytes, platelets, ferritin, lactate dehydrogenase [LDH], C-reactive protein [CRP], procalcitonin [PCT]), serum creatinine (SCr), urea, cholesterol, triglycerides, bilirubin, uric acid, creatine kinase [CK], D-dimer, high sensitivity Troponin I [hsTNI], Brain natriuretic peptide [BNP], arterial blood gas [ABG], thick blood smear, electrolytes and urinary analysis [proteinuria, hematuria, pH, specific gravity]), and radiological (thoracic computerized tomography scan [CT] score) data were collected from the admission. In accordance with the local protocol for COVID-19 patients’ management at the CMK, specific biological parameters such as SCr, CRP, PCT, LDH, troponin and BNP were also systematically sampled on day 3 and 7, and beyond if the hospital admission was prolonged. In addition, we collected information about the treatment received (administration of antibiotics, corticosteroids, mechanical ventilation or hemodialysis), complications, and outcomes during the hospital admission. The date on which a complication was noticed or the treatment was initiated or the one reported as the day of exposure was considered as day 1. Chest CT-scans were performed on a 128 Slice HITACHI. SCr was measured by enzymatic method using a COBAS C 111 Automaton-Roche technology (Mannheim, Germany). Proteinuria and hematuria were assessed immediately at the admission from a sample of a 20 mL of urine by a semi-quantitative and visual method using Combur 10 Test-M urinary strips from Cobas-Roche technology, Switzerland.

### Operational definitions

AKI was defined and staged according to the KDIGO guidelines: Stage 1: increase in SCr by 0.3 mg/dL within 48h or a 1.5–1.9 times increase in SCr from baseline within 7 days; Stage 2: 2.9 times increase in SCr within 7 days; Stage 3: 3 times or more increase in SCr within 7 days or new initiation of renal replacement therapy (RRT) [16]. The lowest outpatient serum creatinine values between 7 and 365 days before Hospital admission was considered as baseline creatinine. If no previous results were available, baseline creatinine was the lowest creatinine value between that calculated from an estimated glomerular filtration rate (eGFR) of 75 mL/min/1.72 m^2^ and creatinine reported on admission. Baseline serum creatinine was available prior to admission only in 92/215 (42.7%) patients. The peak serum creatinine value during hospitalization was used to determine the AKI stage. Urine output criteria to define AKI were not used. The COVID-19 patient discharging criteria were defined by at least two negative RT-PCR-COVID-19 tests (within 48 h) in addition to the absence of fever for three days, and a regression of respiratory symptoms and the chest computer tomography (CT) Scan lesions [17]. Proteinuria and hematuria was defined by the presence of semi-quantitative proteinuria or hematuria on the strip ≥1 cross. Renal recovery was defined according to Acute Disease Quality Initiative (ADQI) consensus as the return of serum creatinine (SCr) within 0.3 mg/dL of baseline SCr [18]. The clinical and radiological severity of patients was assessed on admission using the Sequential Organ Failure Assessment (SOFA) [19] and the chest CT severity score, respectively [20]. Acute respiratory distress syndrome (ARDS) was defined and staged according to the Berlin criteria: mild hypoxemia (200 mm Hg < PaO_2_/FIO_2_ ≤ 300 mm Hg), moderate hypoxemia (100 mm Hg < PaO_2_/FIO_2_ ≤ 200 mm Hg), and severe hypoxemia (PaO_2_/FIO_2_ ≤ 100 mm Hg) [21]. Dyspnea was defined as a subjective experience of respiratory discomfort reported by the patient on admission [22].

### Outcomes

The primary outcome was the occurrence of AKI. The second outcome included need for renal replacement therapy (RRT), AKI recovery at discharge, survival defined as time to death and the vital status until the 48^th^ day.

### Statistical analysis

Data were collected and analyzed using Excel and Stata (Stata Corporation version 15.0, Texas, USA 2017), respectively. Descriptive statistics were presented as mean and standard deviation for quantitative data with Gaussian distribution; median and interquartile range (IQR) for quantitative data with non-Gaussian distribution. Proportions were used for categorical data and percentages are based on the total number of non-missing values. Pearson’s chi-square test or Fisher’s exact test (for small numbers in one or more subgroups) was used to compare these proportions. For continuous variables, the comparisons were made using the student’s t-test (variables normally distributed) or Mann-Whitney’s test (variables not normally distributed). Kaplan Meier curves were used to describe patient survival (time to death). Patients who survived at the end of the study or shifted to another center were censored. A comparison survival curves was made using the Log Rank test. Univariate logistic regression analysis was used to identify factors associated with AKI. In order to control the effect of potential confounding, we further performed multivariable logistic regression analysis (using ascending step-by-step approach) to search factors independently associated with AKI. The adjusted ORs and their 95% CI were calculated finally to determine the degree of association between AKI and the independent variables. Only factors with *p* < 0.05 on univariate analysis were entered into the logistic multivariate regression analysis to define factor associated with AKI. The variance inflation factor (VIF) coefficient was used to identify multicollinearity between factors. Two multivariate mathematical models were used to limit the cumulative effect of independent variables. Multivariable cox regression model was used to identify independent factors associated with mortality. The significance level retained was then *p* < 0.05.

## Results

### Patient characteristics

**Figure 1.**
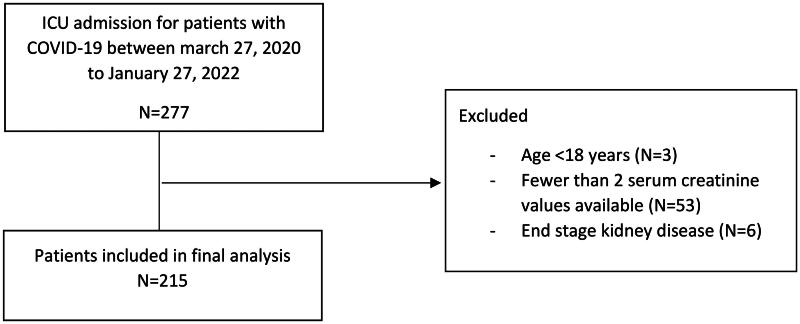
Flow chart of study population selection.

Of 277 consecutive patients with COVID-19 who were admitted to the ICU of the CMK between 27 March 2020 and 27 January 2022, only 215 were included in the final cohort (Figure 1). Portions of this results were presented at the International Society of Nephrology World congress [23]. The mean age and the median (IQR) creatinine of the patients on admission were respectively 58.9 ± 14.9 and 91 µmol/L [74–115]. The majority were male (77%), African ancestry (80.0%), and had hypertension as the main comorbidity in 98 (45.6%) patients. The median(IQR) sofa score of patients and time from hospital admission to discharge or death were respectively 3(2-4) and 10 (9.0–11.0) days. A total of 142/215 (67.3%) had ARDS (mild: 45.1%, moderate: 45.1%, and severe ARDS: 9.8%), 47 (21.9%) were treated with mechanical ventilation, and 37 (17.2%) required vasopressor support during the hospital stay. Norepinephrine was used as the first line vasoactive agent. Urine analysis was available in a total of 170 patients. Proteinuria was the most prominent finding and was documented respectively in 92/170 (54.1%) patients. Hematuria was seen in 32/143 (22.4%) patients. The mean pH and urine-specific gravity were respectively 5.5 ± 0.7 and 1.02 ± 0.007.

### AKI incidence and factors associated

A total of 61/215 (28.4%) patients developed AKI with no difference between the waves (Table 1). Stages 1, 2, or 3 AKI accounted for 39.3%, 11.5%, and 49.2%, respectively. Intermittent hemodialysis was performed on 7.4% of the patients (27.8% of AKI patients) with a high incidence during the 1^st^ wave (*p* = 0.019). The median (IQR) time of initial hemodialysis from hospital admission was 8 (3-10) days.

**Table 1.** Frequency of AKI regarding wave status.

Variables	All Patients (*N* = 215)	1^st^ Wave (*n* = 85)	2^nd^ Wave (*n* = 91)	3^rd^ Wave (*n* = 19)	4^th^ Wave (*n* = 20)	*p*-value
AKI						0.845
No AKI	154 (71.6)	59 (69.4)	65 (71.4)	15 (78.9)	15 (75.0)	
AKI	61 (28.4)	26 (30.6)	26 (28.6)	4 (21.1)	5 (25.0)	
AKI stage						0.815
AKI_1_	24 (11.2)	9 (10.6)	12 (13.2)	2 (10.5)	1 (5.0)	
AKI_2_	7 (3.3)	2 (2.4)	3 (3.3)	0 (0.0)	2 (10.0)	
AKI_3_	30 (14.0)	15 (17.6)	11 (12.1)	2 (10.5)	2 (10.0)	
AKI-D	16 (7.4)	12 (14.1)	2 (2.2)	1 (5.3)	1 (5.3)	**0.019**
AKI Recorvery						0.759
No	41 (67.2)	18 (69.2)	18 (66.7)	2 (66.7)	3 (60.0)	
Yes	20 (32.8)	8 (30.8)	9 (33.3)	1 (33.3)	2 (40.0)	

Data are n (%), or n/N (%). AKI acute kidney injury, AKI-D acute kidney injury requiring dialysis. χ2 test comparing all subcategories.

Comparison between the patients with AKI and those without (Tables 2 and 3) indicated that patients in the AKI group were older with a high proportion of patients with an age greater than or equal to 65 years (52.5% vs 35.1%). Patients with AKI had significantly longer length of hospitalization (11[9–16] vs 9[9–10]), higher N/L ratio (5.0 [5–6] vs 5.0 [3-5]), BNP (293 [59.0-1355] vs 124 [41–520]), SCr at admission (106 [70-156] vs 90 [74–105]). Conversely, they had lower blood arterial pO2 (68.4 ± 21.7 vs 82.1 ± 38.6), BOS (89.3 ± 10.8 vs 93.05 ± 7.5), eGFR at admission (64 [40–91] vs 78 [61–94]), and urinary pH (5.5 ± 0.7 vs 5.8 ± 0.9). AKI patients had a high proportion of dyspnea (52.5% vs 35.1%) and lower proportion of headache (1.6% vs 13%) as a symptom at admission. AKI patients had a high proportion of comorbidity including obesity (32.8 vs 11.0), diabetes (23% vs 11.7%), a higher proportion of proteinuria (65.4% vs 49.2%) and hematuria (36.4% vs 16.2%). Patients with AKI required vasopressors (45.9% vs 5.8%) and mechanical ventilation (54.1% vs 9.1%). Logistic multivariate analysis (Table 4) showed an association between AKI and dyspnea (adjusted odds ratio (OR):2.27 [1.13–4.57] *p* = 0.021), SOFA ≥5 (aOR:3.11[1.29–7.53] *p* = 0.012), AST/ALT ratio (aOR: 1.53 [1.09–1.79] *p* = 0.0015), N/L ratio (aOR:2.09 [1.09–3.20] *p* = 0.016), mechanical ventilation (aOR: 3.20 [1.66–10.51] *p* = 0.005) and Amikacin (aOR: 2.91 [1.37–6.18] *p* = 0.006).

**Table 2.** Baseline demographics, severity-of-illness parameters, and treatment during ICU admission.

Variables	All Patients (*N* = 215)	No AKI (*n* = 154)	AKI (*n* = 61)	*p*-value
Age, years	58.9 ± 14.9	57.8 ± 15.4	61.6 ± 13.2	0.097
Age ≥65 years	86 (40.0)	54 (35.1)	32 (52.5)	**0.015**
Sex males, *n* (%)	166 (77.2)	122 (79.2)	44 (72.1)	0.174
Race African ancestry, *n* (%)	172 (80.0)	121 (78.6)	51 (83.6)	0.264
SOFA	3.0 (2.0–4.0)	2.6 (2.0–3.0)	3.6 (2.5–4.0)	**<0.001**
SOFA ≥5	32 (14.9)	14 (9.1)	18 (29.5)	**<0.001**
Symptoms at admission				
Fever, *n* (%)	147 (68.4)	106 (68.8)	41 (67.2)	0.470
Cough, *n* (%)	106 (49.3)	78 (50.6)	28 (45.9)	0.317
Dyspnea, *n* (%)	86 (40.0)	54 (35.1)	32 (52.5)	**0.015**
Asthenia, *n* (%)	91 (42.3)	66 (42.9)	35 (41.0)	0.463
Headache, *n* (%)	21 (9.8)	20 (13.0)	1 (1.6)	**0.006**
Vomiting, *n* (%)	4 (1.9)	3 (1.9)	1 (1.6)	0.681
Diarrhea, *n* (%)	14 (6.5)	10 (6.5)	4 (6.6)	0.599
Comorbidities				
Hypertension, *n* (%)	98 (45.6)	66 (42.9)	32 (52.5)	0.131
Diabetes, *n* (%)	32 (14.9)	18 (11.7)	14 (23.0)	**0.033**
HIV, *n* (%)	3 (1.4)	2 (1.3)	1 (1.6)	0.635
Obesity, *n* (%)	37 (17.2)	17 (11.0)	20 (32.8)	**<0.001**
Medications before Hospitalization				
ACE inhibitors, *n* (%)	27 (12.6)	18 (11.7)	9 (14.8)	0.343
CCBs, *n* (%)	78 (36.3)	48 (31.2)	30 (49.2)	**0.011**
Diuretics, *n* (%)	32 (14.9)	21 (13.6)	11 (18.0)	0.269
ARBs, *n* (%)	19 (8.8)	12 (7.8)	7 (11.5)	0.271
Beta blockers, *n* (%)	21 (9.8)	12 (7.8)	9 (14.8)	0.100
Medications during Hospitalization				
Corticosteroids, *n* (%)	168 (78.1)	117 (76.0)	51 (83.6)	0.149
Amikacine, *n* (%)	54 (25.1)	26 (16.9)	28 (45.9)	**<0.001**
Vancomycin, *n* (%)	22 (10.2)	6 (3.9)	16 (26.2)	**<0.001**
Iodinated contrast, *n* (%)	107 (53.8)	79 (55.6)	28 (49.1)	0.249
Parameters at admission				
Body temperature (C)	37.2 ± 0.9	37.0 ± 0.8	37.7 ± 1.0	**0.002**
BOS, (%)	91.9 ± 8.7	93.0 ± 7.5	89.3 ± 10.8	**0.007**
Resiratory rate/minutes	28.3 ± 6.2	27.9 ± 5.8	29.5 ± 7.0	0.118
SBP, mmHg	138.9 ± 17.9	138.8 ± 17.5	139.3 ± 18.9	0.899
DBP, mmHg	82.8 ± 13.0	82.8 ± 12.6	82.6 ± 14.0	0.936
Heart rate, bpm	88.0 ± 14.6	86.5 ± 14.5	91.4 ± 14.6	0.124
Dehydratation, *n* (%)	34 (15.8)	20 (13.0)	14 (23.0)	0.058
Arterial blood gaz				
PO2, mmHg	78.2 ± 35.1	82.1 ± 38.6	68.4 ± 21.7	**0.010**
PaO2/FIO2	256 (228–271)	257.5 (227–274)	252 (192–273)	**0.020**
PCO2, mmHg	35.7 ± 8.6	35.5 ± 7.4	36.2 ± 11.0	0.595
SaO2, %	91.6 ± 9.2	92.5 ± 8.7	89.2 ± 10.0	**0.019**
HCO3, mmHg	24.4 ± 4.5	24.6 ± 3.8	23.8 ± 5.9	0.247
Ph	7.4 ± 0.18	7.4 ± 0.05	7.4 ± 0.32	**0.006**
Urinalysis				
Proteinuria				**0.036**
Negative	78 (45.9)	60 (50.8)	18 (34.6)	
Positive	92 (54.1)	58 (49.2)	34 (65.4)	
Proteinuria				**0.011**
Negative	78 (45.9)	60 (50.8)	18 (34.6)	
1+	55 (32.4)	39 (33.1)	16 (30.8)	
2+	22 (12.9)	14 (11.9)	8 (15.4)	
3+	15 (8.8)	5 (4.2)	10 (19.2)	
Hematuria				**0.008**
Negative	111 (77.6)	86 (83.8)	28 (63.6)	
Positive	32 (22.4)	16 (16.2)	16 (36.4)	
Urinary pH	5.7 ± 0.83	5.8 ± 0.85	5.5 ± 0.74	**0.035**
Urine-specific gravity	1.02 ± 0.007	1.02 ± 0.007	1.02 ± 0.007	0.241
CT severity score				**0.033**
Score 0	5 (2.7)	5 (3.8)	0	
Score 1	33 (17.9)	26 (19.8)	7 (13.2)	
Score 2	29 (15.8)	23 (17.6)	6 (11.3)	
Score 3	59 (32.1)	43 (32.8)	16 (30.2)	
Score 4	42 (22.8)	28 (21.4)	14 (26.4)	
Score 5	16 (8.7)	6 (4.6)	10 (18.9)	
Complications				
ARDS, *n* (%)				0.074
ARDS	142 (67.3)	96 (64.0)	46 (75.4)	
No ARDS	69 (32.7)	54 (36.0)	15 (24.6)	
ARDS stage, *n* (%)				0.147
Mild	64 (45.1)	41 (42.7)	23 (50.0)	
Moderate	64 (45.1)	48 (50.0)	16 (34.8)	
Severe	14 (9.8)	7 (7.3)	7 (15.2)	
Rhabdomyolysis, *n* (%)	18 (10.6)	12 (9.8)	6 (12.8)	0.374
Vasopressor use, *n* (%)	37 (17.2)	9 (5.8)	28 (45.9)	**<0.001**
Mechanical ventilation, *n* (%)	47 (21.9)	14 (9.1)	33 (54.1)	**<0.001**
Hospital stay, days	10 (9-11)	9 (9–10)	11 (9–16)	**0.004**

Data are mean ± standard, median (IQR), n (%), or n/N (%). ACE-I: angiotensin converting enzyme inhibitors; ARB: angiotensin receptor blockers; ARDS: acute respiratory distress syndrome; BOS: blood oxygen saturation; Bpm: beats per minutes; CCB: calcium channel blockers; CKD: chronic kidney disease; DBP: diastolic blood pressure, HR: heart rate; RR: respiratory rate; SOFA: sequential organ failure assessment. χ2 test comparing all subcategories.

**Table 3.** Biological and radiological characteristics at ICU admission.

Variables	n	All (n = 215)	No-AKI (n = 154)	AKI (n = 61)	*p*-value
WBC count, ×10^3^ /l	215	6.6 (4.6–8.8)	6.5 (4.6–8.6)	6.7 (5.1–9.8)	0.157
Neutrophils, ×10^3^	215	4.5 (3.1–6.6)	4.3 (2.9–6.2)	5.6 (3.7–7.8)	**0.023**
Lymphocyts, ×10^3^	215	1.2 (0.8–1.6)	1.2 (0.9–1.6)	1.1 (0.7–1.6)	0.131
NLR	215	4.9 (3.1–4.9)	5.0 (3.0–5.0)	5.0 (5.0–6.0)	**0.003**
CRP, mg/l	215	121 (60–217)	114 (53–209)	138 (81–239)	0.136
PCT, ng/ml	215	0.2 (0.1–0.9)	0.2 (0.1–0.8)	0.3 (0.1–1.1)	0.356
Hb, g/dl	215	13.4 ± 1.9	13.5 ± 1.8	13.0 ± 2.2	0.072
Htc, %	215	38.8 ± 5.5	39.1 ± 4.9	38.0 ± 6.7	0.190
Htc/Hb ratio	215	2.9 ± 0.39	2.8 ± 0.43	2.9 ± 0.19	0.215
Platelet count, ×10^3^/l	215	178 (141-238)	179 (140-238)	174 (146–235)	0.533
Ferritin, ng/ml	125	1000 (479–1200)	1000 (470–1200)	1200 (507–1200)	**0.008**
D-dimer, ng/ml	200	1273 (597–3464)	1259 (601–3229)	1384 (404–4374)	0.107
Fibrinogen, ng/ml	150	6.2 ± 2.2	6.3 ± 2.2	5.9 ± 2.1	0.326
HBA1C, %	136	6.9 ± 1.8	6.8 ± 1.7	7.0 ± 1.9	0.611
LDH, UI/L	195	437 (276–628)	434 (273–615)	454 (284–740)	0.270
AST,UI/L	208	53 (32–90)	52 (32–86)	54 (33–92)	0.540
ALT,UI/L	208	38 (26–59)	38 (25–65)	36 (27–55)	0.301
AST/ALT ratio	207	1.4 (1.0–1.9)	1.3 (1.0–1.8)	1.4 (1.1–1.9)	**0.024**
Total Bilirubin,µmol/L	103	8.3 (6.3–12.0)	8 (6–12)	9 (6–12)	0.503
Direct Bilirubin, µmol/l	103	4.6 (3.0–6.7)	4 (3–6)	6 (3–7)	0.558
Thick blood smear positive, n (%)	215	28 (13.1)	23 (15.1)	5 (8.2)	0.128
Malaria parasitemia	28	120 (80–329)	120 (80–3840)	120 (80–6910)	0.583
PTr, %	168	77 (67–93)	78 (68–94)	76 (65–92)	0.204
Ionized calcium, mmol/l	122	1.1 (1.1–1.2)	1.1 (1.1–1.2)	1.1 (1.1–1.2)	0.982
Sodium mmol/l	215	137.3 ± 5.2	137.6 ± 4.9	136.7 ± 4.7	0.326
Potassium, mmol/l	215	3.9 ± 0.53	3.9 ± 0.5	3.8 ± 0.53	0.472
TC, mmol/l	123	4.5 (3.7–5.3)	4 (4–5)	5 (4–6)	0.139
LDLc, mmol/l	123	3.0 (2.3–3.6)	3 (2–4)	3 (2–4)	0.414
HDLc, mmol/l	123	1.0 (0.8–1.3)	1.0 (0.7–1.2)	1.1 (0.9–1.4)	**0.009**
Triglycerids, mmol/l	123	1.5 (1.0–2.1)	2 (1–2)	1 (1–2)	0.425
BUN, mmol/l	215	4.7 (3.6–7.1)	5 (4–6)	6 (4–10)	**0.001**
Creatinin at admission, µmol/l	215	91 (74–115)	90 (74–105)	106 (70–156)	**<0.001**
eGFR at admission, ml/min/1.73m^2^	215	76 (58–94)	78 (61–94)	64 (40–91)	**<0.001**
Peak Creatinin peak, µmol/l	215	104 (83–151)	93 (78–107)	282 (138–479)	**<0.001**
Creatinin at discharge, µmol/l	215	85 (70–114)	78 (66–95)	173 (97–363)	**<0.001**
BUN/creatinin ratio at admission	215	27.2 (21.5–34.6)	27 (21–34)	30 (22–38)	0.226
CK, UI/l	168	182 (89–354)	182 (89–379)	189 (95–325)	0.881
Uric acid, µmol/l	78	347 (253–431)	343 (250–396)	367 (282–478)	0.322
Pro BNP, pg/l	206	149 (45–731)	124 (41–520)	293 (59–1355)	**0.016**
Troponin, ng/l	195	12 (4.5-37.5)	11 (3–28)	20 (7-69)	0.302

Data are mean ± standard, median (IQR), n (%), or n/N (%). Percentage are based on the total number of non-missing values in each category and not necessarily on the total number of participants. *p* values were calculated by Mann–Whitney *U* test or χ2 test ^(2)^, as appropriate. ALT: alanine amino transferase ASAT: aspartate amino transferase; CRP C: reactive protein; Hb: hemoglobin; HDLc: high density Lipoprotein cholesterol; LDH: lactate dehydrogenase; CK: creatinine kinase; NLR: neutrophil-lymphocyte ratio; Pro BNP: brain natriuretic peptide; PTR: prothrombin time ratio; PCT: procalcitonin; TC: total cholesterol; WBC: white blood cell.

**Table 4.** Factors associated with AKI in univariate and multivariate logistic regression analysis.

Variables	Univariate analysis		Multivariate analysis model 1		Multivariate analysis model 2	
	*p*	OR (95% CI)	*p*	aOR (95% CI)	*P*	aOR (95% CI)
Wave 1 vs Wave 3	0.623	1.32 (0.44–4.02)	0.212	1.45 (0.60–1.97)	–	–
Wave 2 vs Wave 3	0.747	1.20 (0.39–3.64)	0.289	1.08 (0.54–2.08)	–	–
Wave 4 vs Wave 3	0.770	0.80 (0.18–3.57)	0.525	0.54 (0.08–3.62)	–	–
Age ≥ 65 years (Yes vs No)	**0.020**	2.04 (1.12–3.73)	0.111	1.77 (0.88–3.56)	–	–
Race (African ancestry vs others)	0.407	1.39 (0.64–3.03)	–	–	0.945	1.34 (0.40–2.69)
SOFA ≥ 5 (Yes vs No)	**<0.001**	4.19 (1.92–9.11)	**0.012**	3.11 (1.29–7.53)	–	–
Dyspnée (Yes vs No)	**0.020**	2.04 (1.12–3.73)	**0.021**	2.27 (1.13–4.57)	–	–
Diabetes (Yes vs No)	**0.040**	2.25 (1.04–4.88)	0.297	1.63 (0.65–4.06)	–	–
Obesity (Yes vs No)	**<0.001**	3.93 (1.89–8.20)	–	–	0.200	1.84 (0.72–4.70)
Amikacin (Yes vs No)	**<0.001**	4.18 (2.17–8.06)	**0.006**	2.91 (1.37–6.18)	–	–
Vasopressor (Yes vs No)	**<0.001**	3.67 (2.90–5.69)	–	–	0.107	2.89 (0.79–10.58)
MV (Yes vs No)	**<0.001**	4.79 (2.59–8.83)	–	–	**0.005**	3.20 (1.66–10.51)
Vancomycin (Yes vs No)	**<0.001**	5.77 (3.24–8.74)	–	–	0.193	2.27 (0.66–7.79)
PO2*	**0.014**	0.98 (0.97–0.99)	–	–	0.621	0.99 (0.98–1.09)
N/L ratio*	**0.012**	1.12 (1.02–1.23)	**0.016**	2.09 (1.09–3.20)	–	–
ASAT/ALAT ratio*	**0.038**	1.35 (1.02–1.79)	–	–	**0.015**	1.53 (1.09–1.79)

Bold values are the statistically significant *p* defining the variables (factors) associated to AKI.

AK: acute kidney injury; ALT: alanine amino transferase, AST: aspartate amino transferase, CCB: calcium channel blockers; CI: confidence intervals MV mechanic ventilation; N: neutrophil; L:lymphocyte; aOR: adjusted odds ratio; OR: odds ratio; SOFA: sequential organ failure assessment.

### AKI and Outcomes

**Figure 2.**
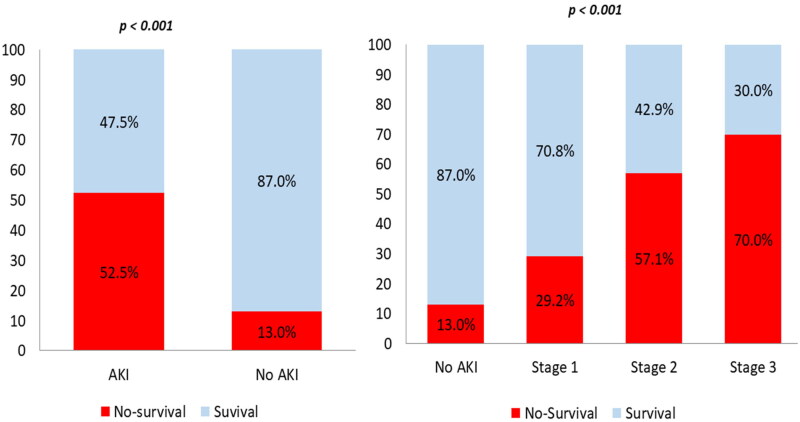
Mortality rate according AKI status and AKI stage.

**Figure 3.**
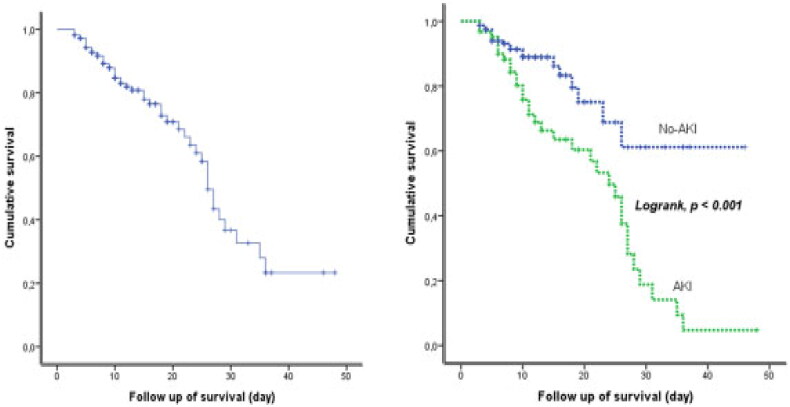
Survival curve of COVID-19 patients study population and according AKI status.

Within our cohort, 52 (24.9%) patients died. The median (IQR) length of stay from hospitalization to death was 10 (7-14) days, while the median (IQR) time from hospitalization to discharge 27 (24–30) days. This mortality (Figure 2) increased with AKI severity (29.2% at stage 1, 57.1% at stage 2, 70.0% at stage 3) and 67.2% of the survivors did not recover kidney function at the end of hospitalization. The median of creatinine value for non-recovered AKI patients at discharge was higher than the recovered group (274.1 [132.6–486.2] vs 97.2[88.4–176.8]) µmol/L. Mortality in the AKI requiring dialysis (AKI-D) group was 94%. In the Kaplan-Meier survival analysis of the two groups based on AKI status (Figure 3), the log-rank test (<0.001) revealed a difference between the groups with a better survival in patients with No AKI (34.7[29.5–39.9)] vs 21.6[18.1–25.1] days). Adjusted Cox regression analysis (Table 5) reported that AKI was independently associated with in-hospital death (HR:2.96 [1.23–4.65] *p* = 0.013).

**Table 5.** Predictors of mortality in COVID-19 patients.

Variables	Analyse univariate		Analyse multivariate	
	*p*	HR (95% CI)	*p*	aHR (95%CI)
Age ≥65 years				
No		1		1
Yes	**0.010**	2.08 (1.19–3.62)	**0.046**	2.46 (1.02–5.93)
BOS**	**0.002**	0.96 (0.93–0.98)	0.716	1.01 (0.96–1.06)
RR**	**<0.001**	1.09 (1.04–1.15)	**0.005**	1.09 (1.03–1.15)
PO2**	**0.002**	0.98 (0.96–0.99)	0.351	0.99 (0.96–1.01)
CRP**	**0.002**	1.04 (1.01–1.06)	**0.045**	1.30 (1.10–1.70)
Ht/Hb ratio**	**0.038**	3.60 (1.07–12.08)	**0.043**	3.02 (1.06–4.26)
AST/ALT ratio**	**0.017**	1.24 (1.04–1.48)	0.575	0.89 (0.62–1.31)
eGFR at admission <60ml/min/1.73m^2^				
No		1		1
Yes	**0.032**	1.84 (1.05–3.20)	0.834	1.09 (0.49–2.42)
AKI				
No		1		1
Yes	**0.001**	2.70 (1.53–4.77)	**0.013**	2.96 (1.23–4.65)
Rhabdomyolysis				
No		1		1
Yes	**0.003**	3.34 (1.49–7.45)	**0.031**	3.48 (1.12–10.81)

Bold values are the statistically significant p defining the variables (factors) associated to mortality aHR adjusted hazard ratio. AKI acute kidney injury. ALT: alanine amino transferase; AST: aspartate amino transferase; BOS: blood oxygen saturation; CRP C:reactive protein; eGFR: estimated glomerular filtration rate; Hb hemoglobin; Ht: hematocrit; HR: hazard ratio RR respiratory rate.

## Discussion

This prospective cohort showed that AKI was common in COVID-19 patients in Kinshasa and was associated with increased mortality. Dyspnea, SOFA score, AST/ALT and N/L ratio, mechanical ventilation and use of Amikacin were associated with AKI. Recovery of kidney function after an AKI episode in COVID-19 patients was very poor.

### Incidence and severity of AKI

Although it is now well established that COVID-19 can induce AKI, the incidence of COV-AKI varies widely depending on the study populations, the AKI definition criteria, the COVID-19 strain, and the improvement in support over time. Although the first Chinese, European, and USA series reported an incidence of AKI which varied between 1 and 42% [24–26], the more recent series report a lower incidence with an average of 29% among patients hospitalized in the USA and in Europe or and 6% in China [27]. In African developing countries, this incidence varied widely from 18% to 50% [28,29]. The incidence in our cohort was 28.4% and increased slightly to 29.6% when considering mainly patients of African ancestry but no statistical difference was observed when comparing ancestry groups.

The relationship between ancestry and the incidence of AKI has been widely reported. People with black ancestry are reported to be at higher risk, and this is ascribed to differences in clinical, socioeconomic, or genetic factors. Grams et al. in 2014, showed that the risk was more related to disparities related to economic status [30]. In their study, by adjusting for the impact of AKI on differences in income and/or insurance, the authors reduced the risk of AKI occurring in African Americans. High-risk alleles of APOL-1 variants were not associated with the risk [30]. The framework of our study allows for this adjustment on the economic status given that all patients admitted to the CMK are covered by some sort of health insurance and therefore shows that improving health coverage in developing countries should have a significant impact on reducing the incidence of AKI. In our cohort, the incidence of AKI-D was high during the first wave and decreased over the time with a slightly increase during the third and fourth wave. This trend was also reported in a retrospective analysis of the incidence rate of hemodialysis unit admission in another COVID-19 center in Kinshasa [23]. This decrease in incidence could be on the one hand the result of a significant reduction in the severity of the disease with time following the acquisition of spontaneous immunity or induced by vaccination, and on the other hand an improvement in the management. As reported elsewhere [31], Otshudiema et al. showed in a retrospective study in the DRC, that the first and second waves of the COVID-19 pandemic in DRC were more severe than the third and fourth waves [14]. Freund et al reported in their cohort a low incidence of acute complication in the vaccinated hospitalized patients [32].

### Factors associated with AKI

The consensus report of the 25th Acute Disease Quality initiative summarized all of the risk factors for AKI-COVID-19 into three broad categories (demographic risk factors, AKI risk factors at admission and during hospitalization) [7]. The main risk factors for AKI in our cohort fell into these different categories.

COVID-19 disease severity is a risk factor of AKI and SOFA score that can be used to measure organ failure is positively correlated with the disease severity. As in previous study, SOFA score was associated with AKI in our cohort [33,34].

The AST/ALT ratio has not only been widely used for the assessment of the progression of liver failure and the prediction of liver fibrosis but also as a factor associated with all-cause and cardiovascular mortality [35]. Its association with renal damage (AKI and/or proteinuria), although little reported during COVID-19, could be the expression on the one hand of the inflammatory syndrome and/or oxidative stress and on the other hand of a hepato-renal syndrome, a consequence of splanchnic vasodilatation from liver dysfunction, as an explanatory factor of AKI in COVID-19 patient [36–38].

As in sepsis, AKI associated with COVID-19 is the consequence of multiple factors, among which is also the dysregulation of the systemic inflammatory response [39]. This is currently well evaluated through the neutrophil/lymphocyte ratio widely used in all medical disciplines as not only a marker of the immune response to infectious diseases but also as a valid index of stress and systemic inflammation [40].

The hemodynamic disturbance associated with mechanical ventilation are those associated with a reduction in cardiac output following the reduction in venous return induced by positive end expiratory pressure (PEEP). Ottolina et al. reported an association between the use of a high PEEP and AKI with a risk 5 times greater [41]. More recently, ventilator-induced lung injury has been proposed as another mechanism of AKI *via* inflammatory crosstalk from the lung to the kidney [42].

The association of the inflammatory marker over time (days 3 and 7) in our study highlighted the possibility of induction of inflammation by AKI (Supplementary Table 1). The association between inflammatory markers and AKI has been widely reported in COVID-19 [43,44] and recent research has shown a bidirectional interplay between AKI and the immune system. Indeed, on the one hand, increased production and decreased clearance of cytokines as well as dysfunction of immune cells, in particular neutrophils, can contribute to immune dysfunction during AKI [45]. On the other hand, during viral infection, white blood cells fight against the offending agent by producing cytokines that stimulate liver to produce C-reactive protein, an inflammatory stress molecule that may promote AKI in COVID-19 with other molecules or pathways such as angiotensin II-associated hypertensive stress diabetes-related metabolic stress, cytokine storm, over-reactive TGF-B signaling, complement activation and lung-kidney cross-talk [45]

AKI associated with aminoglycosides has been also widely reported in the ICU patient population and is not only related to proximal or distal tubular damage but also to glomerular and vascular damage [46,47]. This toxicity is dependent on the cumulative dose and more important in the context of disturbance of the renal hemodynamics [46]. During COVID-19, although AKI associated with aminoglycosides is very little reported [48,49], it is easily understood in the face of disturbances in intra-renal hemodynamics associated with inflammation, hepatic dysfunction, cardiac dysfunction, mechanical ventilation, and significant rhabdomyolysis associated with COVID-19 [50]. The use of amikacin also highlights the possibility of superimposed bacterial infection in COVID-19 patients or the existence of concurrent infectious disease which could explain the occurrence of AKI. These two phenomenon was reported in the literature and suggest the need of early diagnosis to possibly prevent AKI [51,52].

### AKI and outcome

AKI is a strong predictor of mortality in the intensive care unit [53]. This mortality is due on the one hand to metabolic complications specific to renal insufficiency (hyperkalemia, acidosis, volume overload, uremic toxins, etc.) and on the other hand to multi-visceral failure induced by AKI (organ cross-talk) defining AKI as a systemic disease [54,55]. In our cohort, the overall mortality of COVID-19 patients was 25.8%. This mortality increased with AKI status and severity. The death rate of AKI patients was more than 2 time higher than all patients. Considering the AKI patients with hemodialysis, this mortality was more than 3 times higher that AKI stage 1. This trend (higher COVID-19-related mortality with the presence and severity of AKI) has been widely reported by many authors with a higher death rate (69%) than ours in the study by Watanabe et al. an overall mortality of 35% by Hirsh et al. lower than ours [6,56,57] and a mortality of 91% in AKI-D group in the study by Patel et al. similar to the 94% in our study [58]. The disparity found in the literature could be due to differences in the study population as well as the methodologies used. As in most studies, AKI increases the risk of all-cause death by 3 times in our study population [6,57,58].

Renal recovery after an AKI episode depends on the one hand on the nephron reserve and, on the other hand, on the severity of the kidney injury. Nearly 1/3 of patients admitted in the study by Chan et al. had not recovered from their AKI episode [59]. These authors explained these results by the fact that the severity of AKI was greater (as evidenced by the peak in creatinine, and the use of dialysis). This low recovery rate also reported in a Chinese study [60] was contrary to the 80% reported in AKI studies unrelated to COVID-19 [61]. Ng et al. reported a recovery from a post-COVID-19 AKI episode of around 74.1% for stages 1-3 and 66.6% for AKI-D [57].

This disparity also accounts for the difference in the definitions used but also for the follow-up period before discharge. Xu et al. comparing five different definitions of renal recovery, reported that the ADQI criteria may overestimate the extent of renal recovery [62]. Kidney recovery rate is probably higher at longer follow-up as has been demonstrated by Lumlertgul et al. who observed kidney recovery in 90.9% of survivors at 90 days [63] while Schaubroeck et al. only reported 50% at 21 days or at ICU discharge [64]. With a high proportion of stage 3 and the very strict definition of renal recovery according to ADQI, almost 66% of patients with AKI in our cohort had not recovered at hospital discharge with a median time from hospital admission to discharge of 10 (9.0–11.0) days. Racial disparities with black as a risk factor can be another explanation for this poor renal prognosis. COVID-19 can be associated with less renal recovery. Fisher et al. reported 42.3% of renal recovery from COV-AKI vs 68.5% from AKI patients who were negative for COVID-19 [65].

### Strengths and limitations

Our study has some limitations. First, this study was carried out in a single and private center providing specialized tertiary care thus the results cannot be generalized to all COVID-19 patients and can likely represent a selected group and lead to an underestimation of COV-AKI incidence. Second, the small sample size and high level of missing data on some variables (bilirubin, cholesterol, CK, uric acid, hematuria, baseline creatinine) were not sufficiently powered to identify potential associations between variables of interest. Third, the AKI definition was based only on creatinine criteria only. This can bias the study’s estimation of AKI incidence.

Nevertheless, this study has the advantage of being the first one in the DRC to examine epidemiological, laboratory, and radiological data during the course of admission to evaluate some of the risk factors associated with AKI among COVID-19 patients on admission and the temporal changes in laboratory markers from illness onset in patients hospitalized with COVID-19.

## Conclusion

AKI was present in more than a quarter of COVID-19 patients. Inflammatory markers, respiratory disorders and certain medications are associated with AKI. In regard to the outcome, AKI has been associated with an almost threefold increase in overall mortality and nearly 2 third did not recover kidney function at short-term follow-up

## Supplementary Material

Supplemental Material

## Data Availability

The datasets used and/or analyzed during the current study are available from the corresponding author on reasonable request
