# Supplementary material for: Factors associated with acute kidney injury (AKI) and mortality in COVID-19 patients in a Sub-Saharan African intensive care unit: a single-center prospective study
Source: Ren Fail. 2023 Oct 23;45(2):2263583. doi: 10.1080/0886022X.2023.2263583 (PMC11001370; doi:10.1080/0886022X.2023.2263583)
Supplement: Supplemental Material [file IRNF_A_2263583_SM0378.pdf]

Supplementary Table 1. Biological characteristics over the first seven days of ICU admission according to AKI status

Variables	n	All (n=215)	No AKI (n=154)	AKI (n=61)	p-value
**C-reactive protein**					
Day 1, mg/l	215	121 (60-217)	114 (53-209)	138 (81-239)	0,136
Day 3, mg/l	208	70.5 (33.3-146.6)	62 (28-121)	131 (48-236)	**<0.001**
Day 7, mg/l	181	28.0 (11.0-93.5)	23 (8-56)	90 (31-188)	**<0.001**
**Procalcitonin**					
Day 1, ng/ml	215	0.2 (0.1-0.9)	0.2 (0.1-0.8)	0.3(0.1-1.1)	0.356
Day 3, ng/ml	200	0.3 (0.1-1.6)	0.3 (0.1-1.2)	0.7 (0.2-2.4)	0.781
Day 7, ng/ml	171	0.3 (0.1-1.2)	0.1 (0.1-0.7)	0.9 (0.3-3.7)	**0.002**
**Lactate dehydrogenase**					
Day 1, UI/l	195	437 (276-628)	434 (273-615)	454 (284-740)	0.270
Day 3, UI/l	85	436 (343-649)	390 (316-551)	632 (265-907)	0.166
Day 7, UI/l	79	398 (290-581)	330 (273-467)	553 (357-918)	**0.006**
**Brain natriuretic peptide**					
Day 1, pg/l	206	149 (45-731)	124 (41-520)	293 (59-1355)	**0,016**
Day 3, pg/l	76	364 (121-3153)	213 (66-1170)	1155 (284-5310)	**0.011**
Day 7, pg/l	67	581 (161-3199)	288 (112-1314)	1176 (337-5402)	0.150
**Troponin**					
Day 1, ng/l	195	12 (4.5-37.5)	11 (3-28)	20 (7-69)	0.302
Day 3, ng/l	62	30 (11-111)	18 (5-33)	93 (17-151)	**0.024**
Day 7, ng/l	54	17 (5.6-59.4)	8 (2-18)	43 (13-73)	0.354

Data are mean±standard, median (IQR), n (%), or n/N (%). Percentage are based on the total number of non-missing values in each category and not necessarily on the total number of participants. p values were calculated by Mann–Whitney U test or χ2 test, as appropriate.
